# LncRNA as a regulator in the development of diabetic complications

**DOI:** 10.3389/fendo.2024.1324393

**Published:** 2024-02-08

**Authors:** Mengrou Geng, Wei Liu, Jinjie Li, Ge Yang, Yuan Tian, Xin Jiang, Ying Xin

**Affiliations:** ^1^ Jilin Provincial Key Laboratory of Radiation Oncology & Therapy, The First Hospital of Jilin University and College of Basic Medical Science, Jilin University, Changchun, China; ^2^ Key Laboratory of Pathobiology, Ministry of Education, College of Basic Medical Science, Jilin University, Changchun, China; ^3^ Department of Radiation Oncology, The First Hospital of Jilin University, Changchun, China; ^4^ National Health Commission (NHC) Key Laboratory of Radiobiology, School of Public Health, Jilin University, Changchun, China

**Keywords:** long non-coding RNAs, diabetic nephropathy, diabetic retinopathy, diabetic cardiomyopathy, microRNA

## Abstract

Diabetes is a metabolic disease characterized by hyperglycemia, which induces the production of AGEs, ROS, inflammatory cytokines, and growth factors, leading to the formation of vascular dysfunction and target organ damage, promoting the development of diabetic complications. Diabetic nephropathy, retinopathy, and cardiomyopathy are common complications of diabetes, which are major contributors to disability and death in people with diabetes. Long non-coding RNAs affect gene transcription, mRNA stability, and translation efficiency to influence gene expression for a variety of biological functions. Over the past decade, it has been demonstrated that dysregulated long non-coding RNAs are extensively engaged in the pathogenesis of many diseases, including diabetic complications. Thus, this review discusses the regulations of long non-coding RNAs on the primary pathogenesis of diabetic complications (oxidative stress, inflammation, fibrosis, and microvascular dysfunction), and some of these long non-coding RNAs may function as potential biomarkers or therapeutic targets for diabetic complications.

## 1 Introduction

Diabetes mellitus (DM) has become a worldwide epidemic, already affecting one-sixteenth of the global population in 2021, and the prevalence continues to rise annually, with the number of people with the disease expected to reach 783.2 million worldwide by 2045, posing an increasingly serious threat to humanity (1). Type 1 diabetes mellitus (T1DM), an autoimmune disease marked by complete insulin insufficiency as a result of autoimmune β-cell destruction, accounts for approximately 5–10% of all cases of diabetes (2). Furthermore, more than 90% of diabetic individuals have type 2 diabetes mellitus (T2DM), which is characterized by insulin resistance and relative insulin deficiency (2). Therefore, the relative or absolute deficiency of insulin in diabetes induces hyperglycemia and various metabolic signaling disorders that target organs throughout the body and ultimately lead to diabetic complications. Diabetic nephropathy (DN) is one of the most common microvascular complications in diabetic patients and today accounts for almost 40% of all end-stage renal disease (ESRD) (3). The prevalence of diabetic retinopathy (DR) can reach 34.1% and is the leading cause of blindness in adults (4). Diabetic cardiomyopathy (DCM) is difficult to diagnose, has an insidious onset, and is a major cause of death in diabetic patients (5). The burden of the disease and high cost are driven by the presence of chronic diabetic complications, and patients with complications would increase health expenditure by 3.36 times higher compared to those without complications(6).

**Figure 1:**
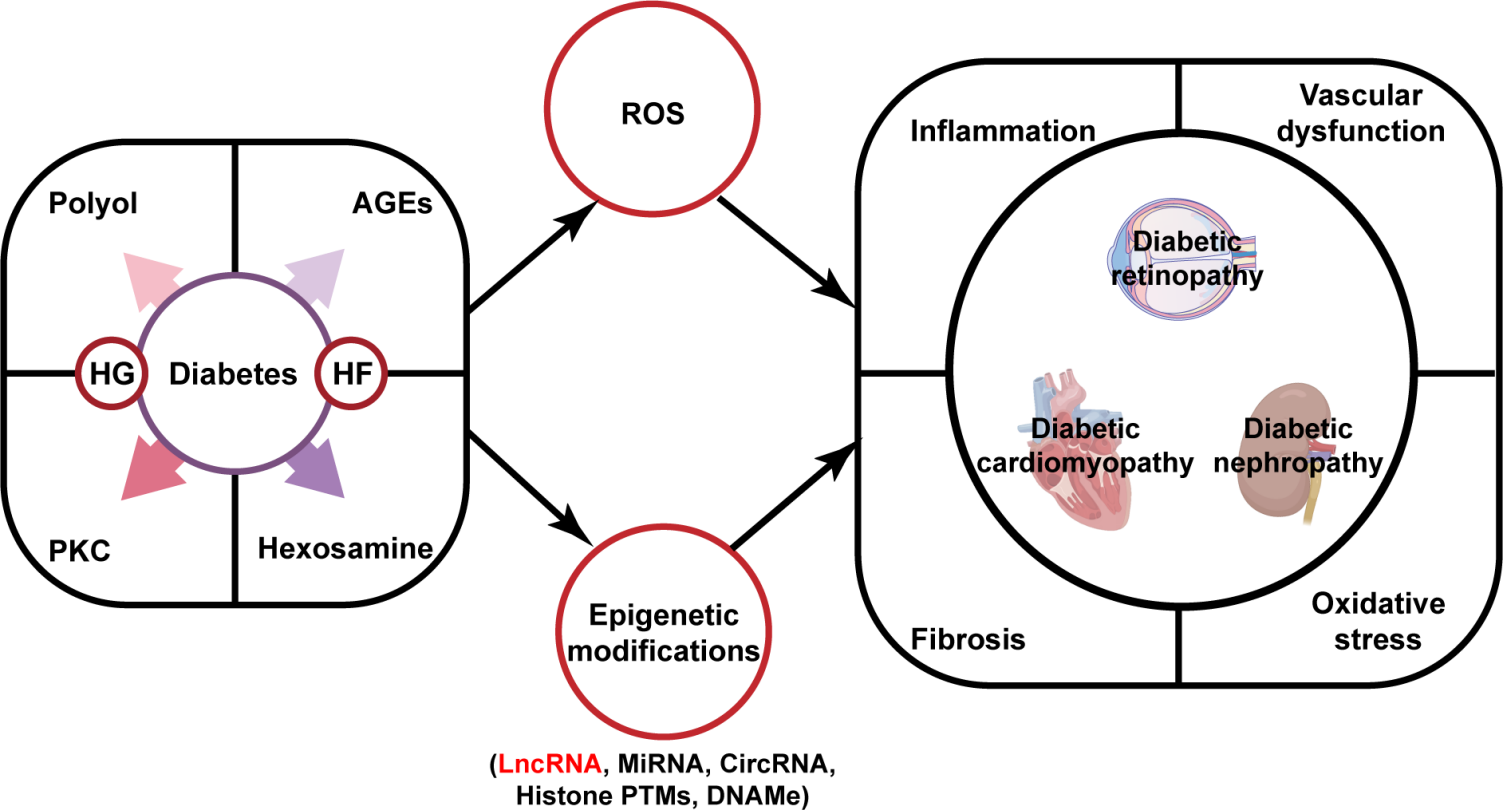
The pathogenesis of diabetic complications and the role of lncRNAs. Diabetes and its attendant metabolic disorders can activate multiple signaling pathways that promote ROS (e.g., superoxide anion) production and dysregulated expression of lncRNAs. These events can lead to the development of pivotal pathological events that consequently have an impact on the progression of DN, DCM, and DR. Abbreviations: HG, high glucose; HF, high fat; AGEs, advanced glycation end products; PKC, protein kinase C; ROS, reactive oxygen species; LncRNA, long non-coding RNA; MiRNA, microRNA; CircRNA, circular RNA; Histone PTMs, histones post-translational modifications; DNAMe, DNA methylation; DN, diabetic nephropathy; DR, diabetic retinopathy; DCM, diabetic cardiomyopathy.

Recent theories on the development of diabetic complications state that multiple cellular pathways are activated by hyperglycemia and dyslipidemia, including activation in polyol pathway flux, intracellular formation of advanced glycation end products (AGEs), expression of the receptor for AGEs and its activating ligands, activation of protein kinase C (PKC) and hexosamine pathway. The activation of these pathways results in production of reactive oxygen species (ROS) (e.g., superoxide anion) and epigenetic changes (DNA methylation, histone modifications, and the expression of non-coding RNAs), which produce growth factors and proinflammatory cytokines that motivate oxidative stress, fibrosis, inflammation, and vascular dysfunction. This leads to pathogenetic alterations and adversely affects endothelial cells, vascular smooth muscle cells (VSMCs), monocytes, and key targets such as retinal cells, cardiomyocytes, and renal cells leading to diabetic complications (7). Although these are common mechanisms in most vascular complications of diabetes, the pathological process and symptoms of the disease can change depending on the target cells and organs. For instance, transforming growth factor β (TGF-β) signaling is activated in a variety of cells in the diabetic kidney and is involved in increased synthesis and deposition of extracellular matrix, ultimately leading to glomerulosclerosis and tubulointerstitial fibrosis. Microvascular dysfunction is a characteristic feature in diabetic retinopathy, with increased microvascular permeability and vascular exudation in the early stages, as well as late neovascular capillary formation. Diabetic cardiomyopathy is seen with inadequate microvascular blood flow and reduced myocardial perfusion, leading to focal necrosis and scar formation, which in turn leads to changes in cardiac structure and function (**Figure 1**). Available anti-diabetic drugs commonly used on the market, such as metformin and sulfonylureas, are presently efficient in regulating hyperglycemia, but they cannot completely prevent the occurrence and progression of its complications. Sometimes these drugs have adverse effects such as liver, heart, and kidney toxicity, hypoglycemia, and gastrointestinal reactions. Consequently, it is essential to understand the underlying molecular mechanisms to develop more effective treatments.

Long non-coding RNAs (lncRNAs) are non-coding RNAs with over 200 nucleotides, which have gained increasing attention from researchers because of their tissue-specific expression patterns and rich regulatory mechanisms. Currently, lncRNAs have been found to regulate gene expression at the transcriptional and post-transcriptional levels. At the transcriptional level, lncRNAs primarily participate in chromatin modification and remodeling, leading to the expression or repression of a large number of genes. Post-transcriptional regulation involves mRNA splicing, translation, and stability. LncRNAs also can regulate protein stability by involving post-translational modifications associated with protein degradation. LncRNAs are increasingly recognized as epigenetic regulators to participate in the development of diabetes and diabetic complications (**Figure 1**) (8). Because of the different onset of T1DM and T2DM, lncRNA can play roles on different targets. In the chronic autoimmune disease of T1DM, it has been recently revealed that viral infections are involved in the attack of pancreatic islet β-cells by immune cells. Evidences showed that lncRNAs *Lnc13* and antiviral response gene inducer (*ARGI*) are upregulated in viral infection and activate the proinflammatory chemokine secretion and antiviral responses (9, 10). In T2DM, lncRNAs are mainly responsible for the insulin resistance. For example, Guo et al. discovered LncRNA *Reg1cp* was mainly expressed in the islet and its mutation was a risk factor for T2DM. Mutant *Reg1cp* increased insulin resistance via inhibiting polypyrimidine tract binding protein 1 (PTBP1) phosphorylation and the PTBP1-AdipoR1 pathway (11). However, lncRNAs also have significant effects on the progression of DM and diabetic complications through regulating the mainly pathogenic progress of oxidative stress, inflammation, cell death, fibrosis, and vascular proliferation. For instance, metastasis associated lung adenocarcinoma transcript 1 (*MALAT1*) was reported to interact with nuclear factor erythroid 2-related factor 2 (Nrf2) as a negative regulator. *MALAT1* ablation activates Nrf2-regulated antioxidant genes expression and reduces ROS accumulation and oxidative stress, resulting in lower inflammation, sensitivity to insulin signaling and improved β-cell function (12). In this paper, biomedical articles published on lncRNAs and diabetic complications in the Pubmed database was searched and the action of lncRNAs in the pathogenesis of DN, DCM, and DR will be reviewed, and this information provides a theoretical basis for the potential use of lncRNAs as therapeutic targets for complications.

## 2 Diabetic nephropathy

DN is one of the primary microvascular complications of diabetes mellitus. Pathologically, DN is characterized by the enlargement of the glomerular mesangial expansion and accumulation of extracellular matrix (ECM) proteins. This leads to glomerulosclerosis and fibrosis in the tubulointerstitial region. In addition, there is damage to the capillary endothelium and the glomerular filtration membrane due to the death of podocytes. All of these factors contribute to kidney dysfunction, which manifests itself early in the form of microproteinuria, reduced glomerular filtration rate, and eventually progresses to end-stage renal disease (ESRD) (13). The current standard treatment for DN involves the use of RAS inhibitors and hypoglycemic agents to manage blood pressure and glucose levels. Unfortunately, these conventional therapies are ineffective in preventing the progression of the disease to ESRD. Many promising novel medications for the treatment of DN have also encountered setbacks in phase 3 clinical trials due to issues such as toxicity. Therefore, there is a growing interest in the development of biomarkers that can predict the early stages of the disease in order to adopt preventative therapy (14). Certain lncRNAs are abnormally expressed in patients with DN and are considered potential biomarkers for its diagnosis. Further research has shown that these lncRNAs have an impact on renal fibrosis and damage to podocytes in diabetic nephropathy, which ultimately affects kidney function (**Table 1**).

**Table 1:** The roles of lncRNAs in DN.

LncRNA	Tissue	Expression	Target	Role in DN	References
*NEAT1*	Renal tissues of DN patients	Up		Promotes proliferation, EMT and deposition of ECM	(15)
	Diabetic mice and diabetic rat renal tissues				(16, 17)
	HG induced mouse mesangial cells		miR-27b-3p/ZEB1; miR-23c; Akt/mTOR		(16, 17)
*ANRIL*	Renal tissues, peripheral whole blood and serum of DN patients	Up		Promotes proliferation and deposition of ECM	(18–22)
	HG induced human renal mesangial cells		miR-15b-5p/WNT2B; miR-98b-5p/NOTCH2		(18, 19)
*TUG1*	Renal tissues of DN patients	Down		Inhibits ER stress and maintains mitochondrial function	(23)
	HG induced human podocytes	Up	CHOP/PGC-1α		(23)
	HG induced mouse podocytes	Down	PGC-1α; ChREBP along with other coregulators enriched at TUG1 promotor		(24, 25)
*MALAT1*	Peripheral whole blood and serum of DN patients	Up		Promotes podocytes oxidative stress, pyroptosis and detachment from the GBM	(26, 27)
	Serum of diabetes-related end-stage renal disease				(28)
	HG induced mouse podocytes		Wnt/β-catenin; miR-200c/Nrf2;		(29, 30)
	HG induced human proximal tubular epithelial cells		Wnt/β-catenin	Promotes EMT	(31)
*PVT1*	Serum of DN patients	Up		Promotes apoptosis	(32)
	Diabetic mice renal tissues				(33)
	HG induced mouse podocytes		EZH2/FOXA1		(33)
*ARAP1-AS2*	Serum of DN patients	Up		Promotes proliferation and EMT	(34)
	HG induced human proximal tubular epithelial cells		ARAP1		(35)
*CASC2*	HG induced human renal mesangial cells	Down	miR-135a-5p/TIMP3	Inhibits proliferation, inflammation, and fibrosis	(36)
Gm4419	HG induced mouse mesangial cells	Up	NF-kB	Promotes inflammation and fibrosis	(37)

NEAT1, Nuclear Enriched Abundant Transcript 1; HG, high glucose; MALAT1, Metastasis Associated Lung Adenocarcinoma Transcript 1; PVT1, Plasmacytoma Variant translocation 1; TUG1, Taurine-Upregulated Gene 1; DN, diabetic nephropathy; EMT, epithelial-mesenchymal transition; ECM, extracellular matrix; ER, endoplasmic reticulum; GBM, glomerular basement membrane; WNT2B, wingless-type family member 2B; NOTCH2, notch homolog 2; TGF-β1, transforming growth factor β1; Nrf2, nuclear factor erythroid 2-related factor 2; CHOP, C/EBP homologous protein; PGC-1α, peroxisome proliferator-activated receptor gamma coactivator 1α; EZH2, zeste homolog 2; FOXA1, forkhead box A1; ARAP1-AS2, ARAP1 antisense RNA2;CASC2, cancer susceptibility candidate 2; TIMP3, Tissue inhibitors of metalloproteinases 3.

### 2.1 Renal fibrosis

Renal fibrosis has been recognized as one of the most crucial processes for the development of DN and is significantly associated with DN prognosis. Anti-fibrotic treatment significantly improves renal function. Renal fibrosis is manifested as excessive deposition of the ECM. It is widely accepted that myofibroblasts play a major role in the synthesis and secretion of ECM under pathological conditions (38). Mesangial cells and renal tubular epithelial cells are considered to be an important precursor cell type of myofibroblasts in DN, transformed into myofibroblasts by epithelial mesenchymal transition (EMT) in response to high sugar stimulation (38, 39). Lately, there is evidence pointing to the involvement of lncRNAs.

Nuclear Enriched Abundant Transcript 1(*NEAT1*) has been reported to dysregulate in DN (15). Previously, it was shown that AKT/mTOR is a key signaling pathway initiated by the kidney in response to high glucose contributing to glomerular hypertrophy (40). *NEAT1* upregulation has positive effects on mesangial cell growth and secretion of ECM by increasing AKT and mTOR phosphorylation levels (16). Moreover, there is evidence that *NEAT1* takes part in renal fibrosis by advancing the EMT process. Zinc finger E-box binding homeobox 1(ZEB1), a key molecule in EMT initiation and activation, is upregulated by *NEAT1* by sponging miR-27b-3p. *NEAT1* deficiency significantly reduces the secretion of EMT proteins (E-calmodulin, N-calmodulin) from mesangial cells (17). It also activates bovine serum albumin (BSA)-mediated EMT and fibrosis in HK-2 cells via the ERK1/2 pathway. Silencing of *NEAT1* reversed renal tubular epithelial cells migration and the expression of mesenchymal markers such as α-SMA and inhibited the transformation of renal tubular epithelial cells into myofibroblasts. And NEAT1 is the most significantly repressed lncRNA in kidney tissue of Klotho (an antiaging protein) overexpressing diabetic mice. These results imply that targeted *NEAT1* implicates the protective effect of Klotho on renal tubular epithelial cell fibrosis and EMT (41). Furthermore, ARAP1 antisense RNA2 (*ARAP1-AS2*) leads to cytoskeletal rearrangement by interacting with ARAP1, and *MALAT1* activates the Wnt/β-catenin pathway to promote the transformation of renal tubular epithelial cells into myofibroblasts (31, 35).

LncRNAs engage in renal fibrosis by promoting ECM secretion. Antisense Non-coding RNA in the *INK4* Locus (*ANRIL*), also known as cell Cycle protein-Dependent Kinase Inhibitor 2B Antisense RNA1 (*CDNK2B-AS1*), is discovered to be elevated in the renal tissues of people with diabetic nephropathy and has been linked to the development of DN via a variety of pathways (18–22). *ANRIL* knockout has a protective on diabetic mouse kidneys, revealing a reduction in urine output and albumin creatinine levels, as well as decreased mesangial matrix depositions and fibronectin levels (42). The underlying mechanism displayed that *CDKN2B-AS1* interference reverses the ECM accumulation and mesangial cell growth by regulating the miR-15b-5p/Wingless-Type family member 2B (*WNT2B*) axis (18). Notch homolog 2 (NOTCH2) is one of the important receptors in the NOTCH pathway, which also mediates renal fibrosis. Xiao et al, display that *NOTCH2* acts as a target of *ANRIL* facilitates apoptosis and fibrosis of high glucose-treated HK-2 cells, and is overturned by miR-98-5p overexpression (21).

Moreover, LncRNA cancer susceptibility candidate 2 (*CASC2*) is reported to exert a protective role in DN by modulating the inflammation. Tissue inhibitors of metalloproteinases 3 (TIMP3) is identified as endogenous specific inhibitors of matrix metalloproteinases in the kidney. *CASC2* functions as competing endogenous RNA (ceRNA) to upregulate TIMP3 expression by sponging of miR-135a-5p and alleviates inflammatory response and fibrosis of mesangial cells (36). *Gm4419* was highly expressed in renal tissues of DN mice and formed positive feedback with p50, the subunit of NF-kB. The pro-inflammatory and fibrosis biomarkers were upregulated in mesangial cells when Gm4419 was overexpressed (37).

### 2.2 Podocytes damage

Podocytes are highly specialized terminally differentiated cells that, together with the glomerular basement membrane (GBM) and endothelial cells constitute the glomerular filtration barrier, which leads to a significant correlation between podocyte damage and the severity of proteinuria. LncRNAs are also discovered to be a partial participant in the podocyte damage in the development of DN.

LncRNA Taurine-Upregulated Gene 1 (*TUG1*) is poorly expressed in the renal tissues of people with DN (23). Recent studies have identified the precise regulation of *TUG1* by high-glucose (HG) environments and the downstream regulatory mechanisms of *TUG1* that link cellular metabolic states to cellular life activities. The study conducted by Long et al, found that HG enhances the transportation of the transcription factor ChREBP and other coregulators, such as MAX dimerization protein (MLX), MAX dimerization protein 1 (MXD1), and histone deacetylase 1 (HDAC1) to the nucleus. These co-regulators are particularly abundant in the *TUG1* promoter and suppress *TUG1* expression (24). *TUG1* exhibits an evident negative effect on the expressions of markers of endoplasmic reticulum stress (ERS) in the cultured podocytes treated with HG, such as eukaryotic translation initiation factor 2α (eIF2), glucose-regulated protein (GRP78), and C/EBP homologous protein (CHOP). *TUG1* significantly enhances peroxisome proliferator-activated receptor gamma coactivator 1α (PGC-1α) expression by deregulating the inhibitory effect of CHOP on PGC-1α. PGC-1α is a transcriptional activator that is significantly associated with mitochondrial morphology and dynamics and plays a protective role in kidney injury. Thus, TUG1 overexpression rescues HG-induced podocyte loss and reduced number the of podocytes (43). Further investigation of the renoprotective mechanism of *TUG1*/PGC1 signaling reveals that PGC1 is necessary for *TUG1* maintenance of the mitochondrial biogenesis, dynamics, redox, and bioenergetics of podocytes, which is partly mediated by negatively regulating the transcription of arginase 2 (AGR2)(25).

*MALAT1* expression is considerably higher in DN patients than in T2DM patients, and it can be utilized to identify DN in conjunction with other biomarkers (ACR, creatinine, and 1-MG) (26, 28). Besides this, *MALAT1* correlates directly with biomarkers of podocyte damage (synaptopodin, podocalyxin), and exerts negative effects upon the podocytes (27). Further research demonstrates that *MALAT1* may play a role in the detachment of podocytes from GBM. P-cadherin is a key component of the slit diaphragm and was found to be associated with podocyte adhesion(44). *MALAT1* is upregulated in the nucleus of high glucose-treated podocytes and is involved in variable splicing of β-catenin. *MALAT1* reduction increases P-cadherin levels and reduces podocyte damage (29). Moreover, the knockdown of *MALAT1* protects MPC-5 cells from HG-induced pyroptosis and oxidative stress through upregulation of the *Nrf2* expression (30).

Plasmacytoma Variant translocation 1 (*PVT1*) is the first lncRNA suspects to be involved in kidney diseases, and two studies in 2007 reported the role of *PVT1* in mediating susceptibility to ESRD caused by type 2 and type 1 diabetes, providing a rationale for *PVT1* as a candidate gene for ESRD (45, 46). There is strong evidence from subsequent studies that *PVT1* is essential in renal parenchymal cell injury and increases in the serum of patients with diabetic nephropathy (32, 47). Recently, it has been shown that *PVT1* localizes to the nucleus of podocytes and silences forkhead box A1(*FOXA1*) expression by recruiting zeste homolog 2 (EZH2) to the *FOXA1* promoter region. FOXA1 is a transcription factor that has been identified to regulate apoptosis through inducing the expression of *Bcl-2*. *PVT1* silencing or overexpression of *FOXA1* attenuates podocyte apoptosis *in vitro* and *in vivo* (33).

The above lncRNAs summarized in **Table 1** are dysregulated in DN and have been found to play a role in DN by regulating multiple pathological processes such as mesangial cell proliferation, ECM deposition, podocyte detachment, and apoptosis, and may be useful as new promising therapeutic target.

## 3 Diabetic cardiomyopathy

**Figure 2:**
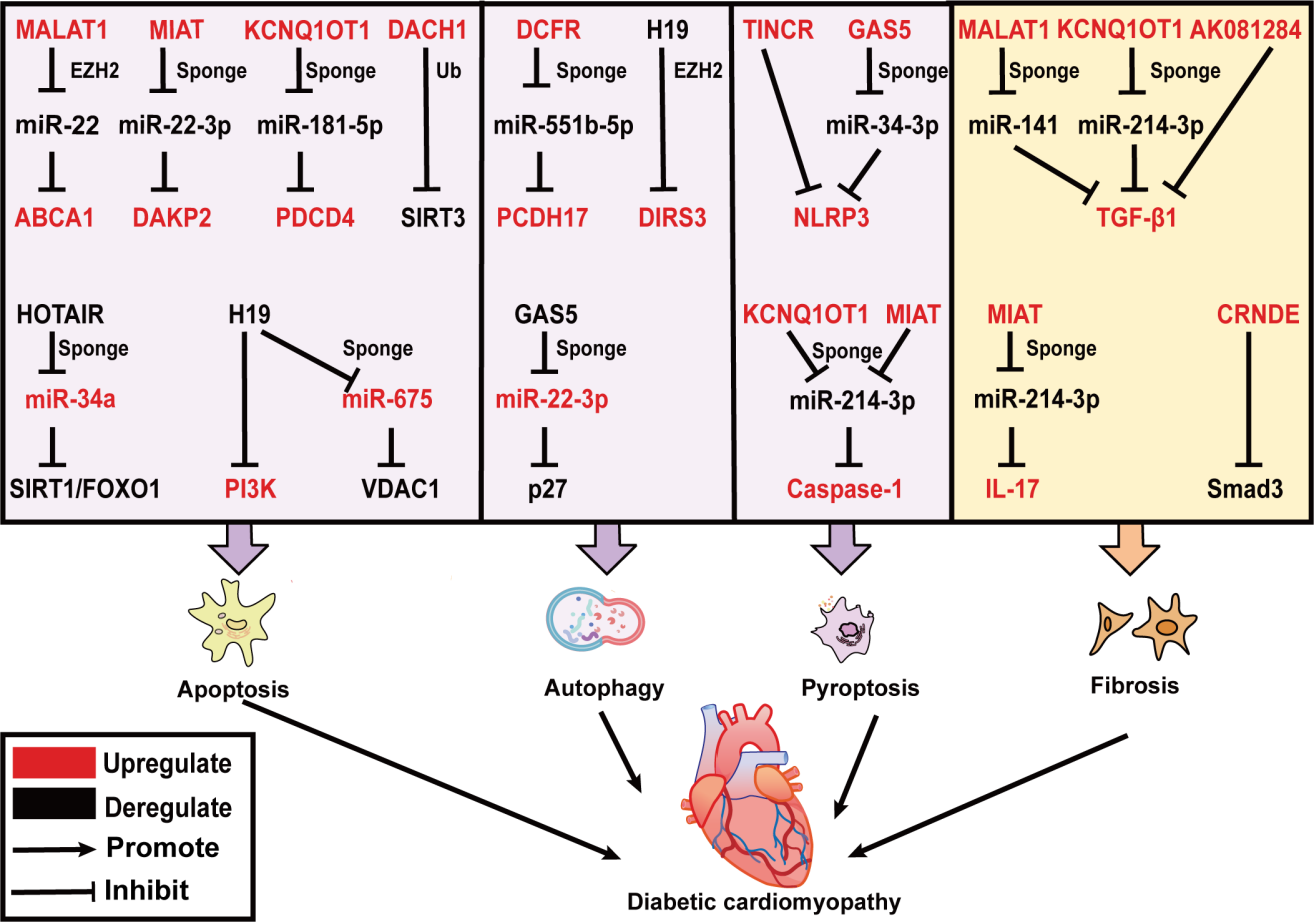
LncRNAs effect on DCM by regulating cardiac apoptosis, autophagy, pyroptosis and fibrosis. LncRNAs are mainly involved in three modes of death including apoptosis, autophagy, and pyroptosis in DCM. *MALAT1, MIAT, KCNQ1OT1*, and *DACH1* promote cardiomyocyte apoptosis, while *HOTAIR* and *H19* prevent cardiomyocyte apoptosis. *DCRF* and *H19* inhibit, and *GAS5* promotes cardiomyocyte autophagy. *TINCR, GAS5, KCNQ1OT1*, and *MIAT* trigger cardiomyocyte pyroptosis. In addition, *KCNQ1OT1, MALAT1, AK081284*, and *MIAT* induce TGF-β1 secretion and cardiac fibrosis. *CRNDE* inhibits Smad3 phosphorylation and suppresses cardiac fibrosis. The upregulated lncRNAs, miRNAs and target genes in DCM are represented in red color; while those downregulated are represented in black color. Abbreviations: *MALAT1*, Metastasis Associated Lung Adenocarcinoma Transcript 1; *MIAT*, Myocardial Infarction Associated Transcript; *KCNQ1OT1*, *KCNQ1* Opposite Strand/Antisense Transcript 1; *DACH1*, Dachshund Family Transcription Factor 1; *GAS5*, Growth Stabilization Specific Transcript; *TINCR*, Terminal Differentiation-induced NcRNA; *CRNDE*, Colorectal Neoplasia Differentially Expressed; Ub, Ubiquitination; ABCA1, ATP-binding cassette transporter A1; DAPK2, death-associated protein kinase 2; PDCD4, programmed cell death protein 4; SIRT3, sirtuin 3; EZH2, zeste homolog 2; NLRP3, NOD-like receptor family pyrin domain containing 3; TGF-β, transforming growth factor β; VDAC1, voltage dependent anion channel 1; IL-17, interleukins-17.

DCM is one of the most serious diabetic complications and was first identified in 1972. Rubler et al, reveal that this disease occurs in diabetic patients who develop heart failure in the absence of coronary artery disease, hypertension, and valvular heart disease (48). In the early stage, it is characterized by hyperglycemia caused by insulin resistance and increased free fatty acid levels, and only diastolic dysfunction has not yet appeared in the structural and morphological changes of cardiomyocytes. Later, with metabolic disorders and long-term neurohumoral abnormalities, myocardial cell death increases and interstitial fibrosis impairs systolic and diastolic function. Further decline in cardiac compliance in late DCM increases the prevalence of heart failure (49). LncRNAs have been found to regulate different forms of death (apoptosis, pyroptosis, and autophagy) as well as fibrosis in DCM (**Figure 2**).

### 3.1 Apoptosis

Increased cardiac apoptosis has been indicated as a leading cause of a major risk factor for the development of DCM (50), as supported by the evidence that cardiomyocyte apoptosis is 85 times more prevalent in the biopsied cardiac tissue of DCM patients than in control non-diabetic hearts. Some lncRNAs have been reported to be upregulated in DCM mice and promote apoptosis in cardiomyocytes. *MALAT1* knockdown can restore cardiac function and suppress cardiomyocyte apoptosis by inhibiting ATP-binding cassette transporter A1 (*ABCA1*) expression and raising miR-22 expression. In this study, *MALAT1* can interact with EZH2 and recruit it to the miR-22 promoter region, where it might epigenetically suppress miR-22 transcription in cardiomyocytes (51). MiR-22-3p is directly targeted with Myocardial Infarction Associated Transcript (*MIAT*) in an AGO2-dependent manner. *MIAT* increases the death-associated protein kinase 2 (DAPK2) levels via sponging miR-22-3p, promoting apoptosis in cardiomyocytes in diabetic rats (52). Recently programmed cell death protein 4 (PDCD4) is considered to be involved in the progression of diabetic cardiomyocytes (53), which serves as a tumor suppressor in prior studies (54). *KCNQ1* Opposite Strand/Antisense Transcript 1 (*KCNQ1OT1*) can serve as a ceRNA for miR-181a-5p to regulate the expression of *PDCD4*, which contributes to the inflammatory response and apoptosis in human cardiomyocytes under HG conditions (55).

Mitochondrial dysfunction and ROS are of great interest to trigger apoptosis, and it is recognized that lncRNAs are engaged in this process. SIRT3 can enhance the capacity of mitochondria to eliminate overproduction of ROS by deacetylating and activating superoxide dismutase (SOD). SIRT3 belongs to the sirtuin (SIRT) family, which is a primary mitochondrial deacetylase. In neonatal mouse ventricular cardiomyocytes (NMVCs) exposed to HG conditions, lncRNA Dachshund Family Transcription Factor 1 (*DACH1*) overexpression notably increases ROS accumulation and apoptosis by promoting SIRT3 ubiquitination (56). In contrast, HOX Transcript Antisense Intergenic RNA (*HOTAIR*) alleviates oxidative stress and myocardial death of DCM via sponging miR-34a and activating the SIRT1/FOXO1 pathway (57), is specifically downregulated in DCM patients and serves as a promising biomarker for DCM (58). Furthermore, ROS accumulate in the endoplasmic reticulum (ER), increasing the number of misfolded proteins and finally causing ERS. *H19* plays a protective role in the progression of DCM. It restores left ventricular dysfunction in the heart of STZ‐induced diabetic mice, as well as under HG culture, suppresses ERS-elicited myocardial apoptosis by activating PI3K in HL-1 cells (59). Additionally, voltage dependent anion channel 1 (VDAC1) plays a crucial role in mitochondria-mediated apoptosis. *H19*-derived miR-675 (60), through downregulation of its target VDAC1, represses hyperglycemia-mediated oxidative stress and apoptosis in cardiomyocytes (61).

### 3.2 Autophagy

Autophagy is a ubiquitous process, that is responsible for eliminating harmful protein aggregates, intracellular pathogens, and superfluous proteins by the lysosomes (62). Autophagy has been controversial in the sense of being beneficial or disadvantageous to the heart. In general, appropriate levels of autophagy protect cardiomyocytes from apoptosis, while its excessive activation leads to autophagic cell death (50). This accounts for the fact that the effect of lncRNA-regulated autophagy on cardiac function is also two-sided. Growth Stabilization Specific Transcript (*GAS5*) promotes autophagy to ameliorate cardiomyocyte hypertrophy, myocardial fiber breakage, and mitigated synthesis of collagen. Mechanistically, *GAS5* positively regulates *p27* gene by binding with miR-221-3p and raising the levels of p62 and LC3B II, reversing the inhibition of autophagy in HG-processed H9c2 cells (63, 64). Conversely, *DCRF*, a newly discovered lncRNA, is boosted in the myocardium of STZ‐induced diabetic mice (65). It is mainly expressed in cardiomyocyte cytoplasm and is directly targeted at miR-551b-5p. Protocadherin 17 (*PCDH17*), which belongs to the protocadherin gene family, has been evidenced to be linked with the activation of autophagy in cancer cells (66, 67). *DCRF* can enhance *PCDH17* expression by sponging miR-551b-5p, thus promoting autophagy in cardiomyocytes of STZ‐induced diabetic rats. Reduced expression of *DCFR* alleviates myocardial fibrosis and restores cardiac function. Likewise, *H19* overexpression inhibited autophagy to improve cardiac function in T1DM rats. *H19* can interact with enhancer of EZH2 to exert effects on DIRAS family GTPase 3 (DIRAS3) transcription in cardiomyocytes, which results in epigenetically suppressing DIRAS3 and activating mTOR signaling to inhibit autophagy (68).

### 3.3 Pyroptosis

Although both pyroptosis and apoptosis are forms of programmed death, pyroptosis leads to the breakdown of the plasma membrane and rapid release of large amounts of inflammatory contents into the extracellular compartment to induce inflammation, whereas apoptosis is immunologically silent and the contents of the dying cell are contained within apoptotic bodies (69). The canonical pathway of pyroptosis is through the activation of caspase1 by NOD-like receptor family pyrin domain containing 3 (NLRP3) inflammasome, which converts interleukins 1beta and 18 (IL-1β and IL-18) precursor into mature forms while cleaving gasdermin D (GSDMD), forming pores in the plasma membrane, and resulting in cell swelling and lysis (70).

According to the present research, by regulating NLRP3 and caspase-1, lncRNAs have a significant impact on the emergence of DCM. Cardiac dysfunction in diabetic rats is significantly reversed by MCC950 (NLRP3 inhibitor). Similarly, HG treated neonatal rat ventricular myocytes and H9c2 cells exhibit characteristic pyroptosis promoted by elevating Terminal Differentiation-induced NcRNA (*TINCR*). RNA pull-down assays reveal that *NLRP3* mRNA is prominently enriched by *TINCR*, and *TINCR* knockdown accelerates *NLRP3* mRNA degradation in cardiomyocytes to inhibit pyroptosis (71). *GAS5*, acting as a ceRNA and being downregulated in DCM mice, forms a feedback loop with the *NLRP3* negative regulator AHR and miR-34-3p to alleviate pyroptosis in HL-1 cells (72). The expression of lncRNA *KCNQ1OT1* is found to rise in HG-induced cardiac fibroblasts and diabetic mice. The binding of *KCNQ1OT1* with its target of miR-214-3p disrupts the interaction of miR-214-3p with caspase-1, leading to the initiation of primary mouse cardiac fibroblast pyroptosis (73). Similarly, bioinformatic prediction analysis indicates that miR-214-3p potentially contains both *MIAT* and caspase-1-binding sites. Silencing *MIAT* by a small interfering RNA suppresses the expression of caspase-1, IL-1β, IL-18, and GSDMD, and ameliorates cardiac pyroptosis in C57BL/6 mice (74).

### 3.4 Fibrosis

Myocardial cell death stimulates inflammation and subsequent myofibroblasts activation, leading to the formation of reparative fibrosis. Fibrosis is one of the key factors in the development of DCM, leading to ventricular remodeling, contractile failure, and diastolic dysfunction. Cardiac fibroblasts (CFs) converted to myofibroblasts (MFs), which display boost levels of collagens and alpha‐SMA (a marker of CFs activation into MFs), are required for cardiac fibrosis (75). TGF-β1/Smads signaling pathway plays a crucial role in the transformation of CFs into MFs, and it significantly promotes myocardial fibrosis. A growing body of evidence suggests that lncRNAs take part in the dysregulation of the TGF-β1/Smads signaling pathway in DCM. LncRNA *MALAT1* directly increases the TGF-β1 expression in HG-treated CFs by acting as a miR-141 sponge. Ablation of *MALAT1* alleviates cardiac interstitial fibrosis and enhances cardiac contractility in diabetic mice (76). Interleukins-17 (IL-17) protein expression is upregulated in HG-treated fibroblasts. IL-17 ultimately promotes fibroblast proliferation and secretion of TGF-β1 and α-SMA through increased expression of lncRNA *AK081284*, which promotes fibrosis (77). *MIAT* as an upstream molecule of IL-17, is responsible for increasing IL-17 production by sponging miR-214-3p in cardiomyocytes (78). *KCNQ1OT1* also targets miR-214-3p and attenuates the inhibition of TGF-β1/Smads pathway activation by miR-214-3p (73). Zheng et al, demonstrate that Smad3‐Colorectal Neoplasia Differentially Expressed (*CRNDE*) negative feedback loop exerts in mouse neonatal CFs. LncRNA *CRNDE* can compete with TGF-β1 to bind Smad3 through rSBEs, thereby preventing TGF-β-mediated phosphorylation of smad3. Smad3, in turn, activates *CRNDE* transcription. Accordingly, silencing *CRNDE* elevates CFs collagen deposition and aggravates left ventricular ejection fraction (79).

Overall, above mentioned lncRNAs are involved in regulating different cell death pathways and fibrosis in DCM, which is summarized in **Figure 2**. Some lncRNA can even directly link cell death to fibrosis, for illustration, *MIAT* and *KCNQ1OT1* can both bind miR-214-3p through the ceRNA mechanism and promote pyroptosis and fibrosis in DCM (73, 78). These important lncRNAs have the potential to become new targets for the treatment of DCM in the future.

## 4 Diabetic retinopathy

DR is a frequent consequence of diabetes, both type 1 and type 2. The severity of DR is influenced by age and the progression of the disease (80). Prolonged oxidative stress, release of pro-inflammatory factors and vascular endothelial growth factor (VEGF) induced by DM damage neurovascular and endothelial cells. This results in increasing vascular permeability, angiogenesis, and impairment of the blood-retinal barrier (BRB). DR has historically been divided into two types: non-proliferative diabetic retinopathy (NPDR) and proliferative diabetic retinopathy (PDR) (81). Microaneurysms and blood vessel leakage are features of NPDR in the early stages, which are followed by swelling and blood vessel obstruction in the later phases. PDR involves the growth of new blood vessels behind the retina and vitreous. VEGF is a target for treatment, and it can lead to regression of vascular lesions and improvement in the severity of DR. However, VEGF treatment requires frequent administration and is most effective in advanced disease stages. This means that new therapeutic targets other than VEGF need to be found (82). The retina is a neural tissue and neurodegeneration has been demonstrated to occur earlier than vascular abnormalities both in animal models and DR patients(83). Researchers have discovered that lncRNAs play a critical role in the development of retinal neurodegeneration and vascular dysfunction. They also have the potential to be innovative treatments.

### 4.1 Diabetic retinal neurodegeneration

Müller cells are the major glial cells in the retina, spanning the entire retina and mediating neuronal and vascular interactions, thus dominating the retina (83). Recent studies have demonstrated that Müller cells are crucial for the development of DR and that may be connected to the proinflammatory cytokines released from them (84). Zhang et al, suggest that C-myc impacts the release of proinflammatory cytokine by mediating *MIAT*/thioredoxin-interacting protein (TXNIP) pathway. C-myc binds to the *MIAT* promoter and up-regulates it expression which is markedly promoted by HG stimulation. Furthermore, *MIAT* binding to TXNIP protein restrains TXNIP ubiquitination degradation. Previous studies have suggested that TXINP leads IL-1β maturation and inflammation during DR development. As a result, *MIAT* silence diminishes the effects of HG on the release of IL-1β, tumor necrosis factor-alpha (TNF-α), and interleukins-6 (IL-6) from Müller cells, and C-myc over-expression abrogates the impact (85). LncRNA *OGRU* is a newly identified transcript that is found to be markedly up-regulated in serum samples of diabetic patients with DR and plays a strong role in regulating inflammation and oxidative stress. *OGRU* silencing restores Nrf2 protein levels and inhibits nuclear factor kappa-beta (NF-kB) activation in DR rat retinal tissue. *OGRU* over-expression and miR-320 knockdown can increase ROS production by restraining *Nrf2* activation and are reversed through decreasing ubiquitin-specific protease14 (*USP14*) expression. *USP14* deletion also greatly limits the function of IkBa ubiquitination to accelerate NF-kB activation. To further explore the potential of *OGRU* as a therapeutic target, intraocular injection of *OGRU* shRNA in diabetic rats is found to inhibit *OGRU* expression in animals and improve neuronal survival and glial activation. *OGRU* is also involved in angiogenesis and vascular leakage in DR progression, marked by the release of VEGF and TGF-β1 from Müller cells (86).

**Figure 3:**
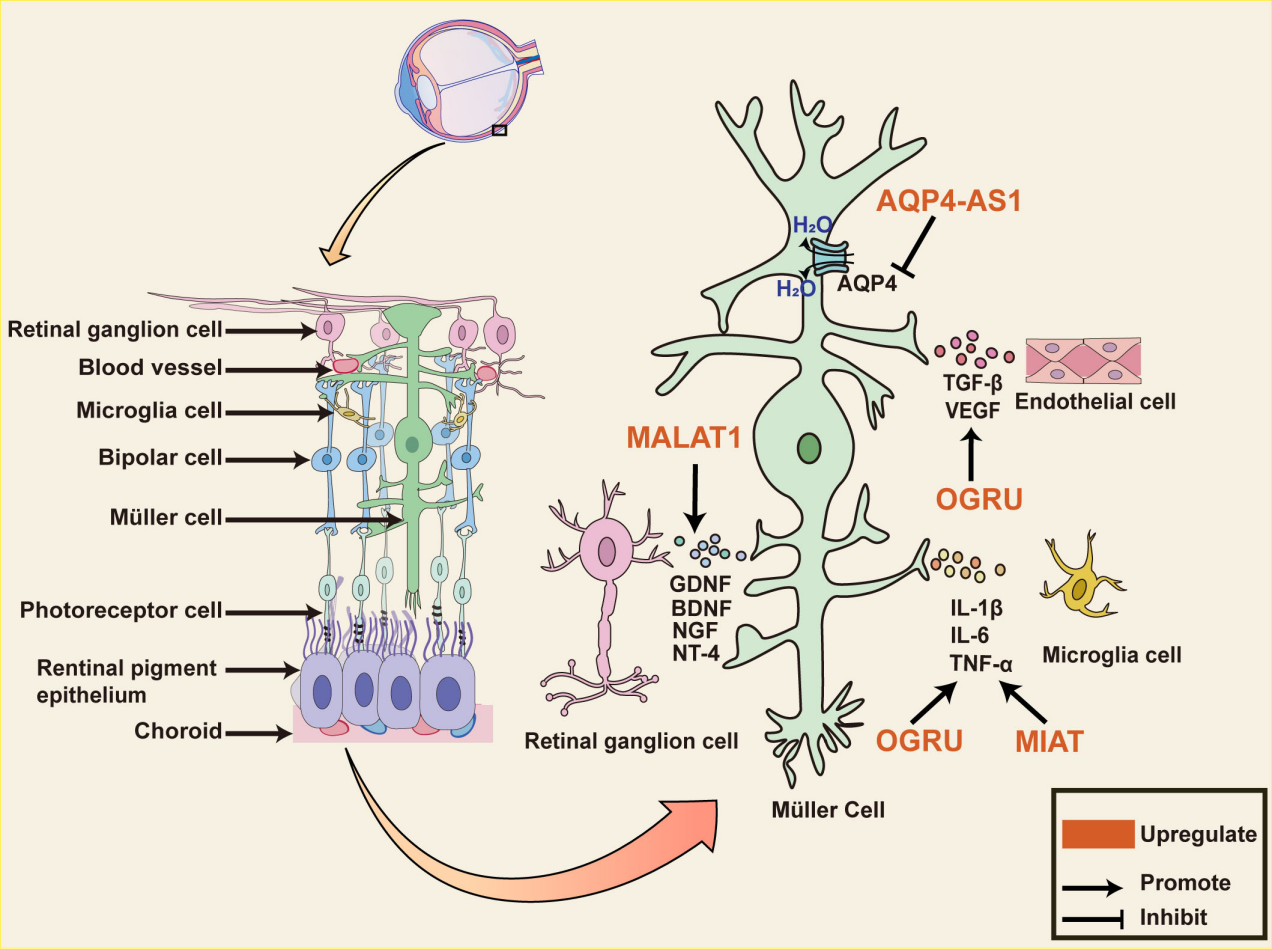
The role of lncRNAs in diabetic retinal neurodegeneration. The diagram shows Müller cells span the entire retina and interact with almost all cells within the retina. Dysregulated lncRNA in Müller cells impacts various pathophysiological events in diabetic retinopathy. *MIAT* and *OGRU* promote the release of pro-inflammatory mediators such as TNF-α, IL-1β, IL-17, and IL-6 from Müller cells. *OGRU* also promotes the release of VEGF and TGF-β1, which are involved in angiogenesis and vascular leakage during DR progression. *MALAT1* upregulates the expression of neurotrophic factors, including GDNF, NT-4, BDNF, and NGF in the retina of optic nerve transection rat, decreasing the number of apoptotic RGCs. AQP4 is the major water channel protein of the central system and is negatively regulated through *AQP4-AS1* in glucose-induced human Müller cells. *AQP4-AS1* silencing reverses Müller cells and RGC cell apoptosis, endothelial cell proliferation, and migration, improving retinal functions. Abbreviations: *MIAT*, Myocardial Infarction Associated Transcript; *MALAT1*, Metastasis Associated Lung Adenocarcinoma Transcript 1; *AQP4-AS1*, *AQP4* Antisense RNA 1; DR, diabetic retinopathy; RGCs, retinal ganglion cells; GDNF, Glial cell-derived neurotrophic factor; BDNF, brain-derived neurotrophic factor; NGF, neurotrophin nerve growth factor; NT-4, neurotrophin-4; VEGF, vascular endothelial growth factor; TGF-β, transforming growth factor β; IL-6, interleukins-6; TNF-α, tumor necrosis factor alpha; IL-1β, interleukins 1beta.

*MALAT1* has a protective effect on DR. *MALAT1* knockdown inhibits Müller cell viability *in vitro* and *in vivo*. Interestingly, the rate of retinal ganglion cells (RGCs) apoptosis is significantly decreased when co-cultured with Müller cell by the releasing of neuroprotective factors, glial cell-derived neurotrophic factor (GDNF), brain-derived neurotrophic factor (BDNF), neurotrophin nerve growth factor (NGF) and neurotrophin-4 (NT-4), and this protective effect is weakened by MALAT1 silencing (87). Aquaporin-4 (AQP4) is the major water channel protein of the central system and is involved in water crossing the blood-brain barrier (88). *AQP4* Antisense RNA 1 (*AQP4-AS1*) is transcribed from the antisense strand of the *AQP4* gene, and is positively regulated in the aqueous humor of diabetic patients. *AQP4-AS1* negatively regulates *AQP4* mRNA in glucose-induced human Müller cells and diabetic retinas. Under the context of high glucose, *AQP4-AS1* silencing reverses human Müller cells apoptosis, RGC cell damage as well as the proliferation and migration of endothelial cells co-cultured with Müller cells. Intravitreal injection of *AQP4-AS1* shRNA in diabetic mice silences its expression, improves retinal dysfunction, and attenuates vascular leakage (89). This suggests that Müller cells play an important role in DR neurovascular crosstalk and dysregulation, and then lncRNAs, which have an important regulatory role in it, are an option for therapeutic targets (**Figure 3**).

### 4.2 Diabetic retinal vascular disease

Normal connections between endothelium in the retinal microvascular system are essential for maintaining vascular function. Vascular endothelial (VE)-calmodulin is a key molecule that mediates interendothelial cell junctions (90). LncRNAs modify VE-calmodulin production through a rich mechanism. Highly *HOTAIR* expression has been shown in DR patients in several studies and has been indicated as an important epigenetic mediator in vascular dysfunction (91). *HOTAIR* acts as a scaffold for lysine demethylase 1A (LSD1) and represses VE-cadherin transcription by decreasing H3K4me3 levels on its promoter (92). *MALAT1* and VE-cadherin are up-regulated while miR-125b is down-regulated in human retina microvascular endothelial cells (hRMECs) treated with HG. *MALAT1* can competitively bind to miR-125b against VE-cadherin at the site of the 3’-untranslated region (3’-UTR), leading to the up-regulation of VE-cadherin(93). Protein kinase C β (PRKCB), a serine-threonine kinase, ubiquitinates VE-calmodulin leading to increased endothelial permeability in retinal vasculature (94). Vascular endothelial‐associated lncRNA‐2 (*VEAL2*) was identified as a novel lncRNA expressed in human umbilical vein endothelial cells (HUVECs). It compete with DAG for binding to the C1 structural domain of PRKCB2 leading to its activation. PRKCB2 translocation to the cell membrane is inhibited by *VEAL2* overexpression and is observed mainly in the cytoplasm, thereby partially reversing endothelial permeability (95).

VEGF has been used as an anti-vascular proliferation target and applied in the clinical treatment of DR, but the efficacy has not been satisfactory. *VEGF-A* mRNA and protein levels are changed under the regulation of *HOTAIR*. *HOTAIR* not only alters *VEGF-A* epigenetic activation but also forms a complex with LSD1 to increase hypoxia inducible factor 1 subunit alpha (HIF-1α) production and promote HIF-1α-mediated transcriptional activation of *VEGF-A* (92). *MALAT1* can likewise directly promote the expression of HIF-1α and VEGF-A through sponging miRNA. According to recent research, HIF1A antisense RNA 2 (*HIF1A-AS2*), the antisense transcript of *HIF-1*, is strongly and positively linked with HIF-1α and VEGF and is higher in peripheral blood in NPDR patients as well as in those with proliferative diabetic retinopathy (PDR) (96). Further explore the therapeutic potential of lncRNA-targeted *VEGF* against microvascular proliferation *in vivo*. In fibrovascular membranes (FVMs) of PDR patients, the co-expression of lncRNA Testis Development Related Gene 1 (*TDRG1*) and *VEGF* around the vessels is observed with immunofluorescence staining. Knockdown of *TDRG1* notably represses the HG-induced *VEGF* expression, resulting in levels close to normal. *TDRG1* silencing rescues hyperglycemia-induced HREC dysfunction, including reducing cell proliferation ability, improving HREC leakage, inhibiting cell migration, and maintaining the tube network formation (97). More and more lncRNAs have been shown to modulate *VEGF* (**Table 2**.). Among them *MALAT1*, Urothelial Carcinoma-Associated 1 (*UCA1*)*, TUG1*, and *linc00174* control VEGF production by regulating various miRNAs via the ceRNA pathway, which suggests that these lncRNAs could serve as potential targets for treating vascular proliferative imbalance in DR patients(98–101).

**Table 2:** LncRNA regulation on VEGF is involved in the progression of DR.

LncRNA	Tissues	Expression	Target	Role in DR	References
*HOTAIR*	Serum samples and VH from proliferative DR Diabetic rats retinal Diabetic mouse retinal HG-stimulated HRMECs HG-stimulated mRECs	Up	LSD1/HIF-1α/VEGF-A, LSD1/VE- cadherin	Promotes angiogenesis and increases endothelial permeability	(91, 92)
*MALAT1*	HG-stimulated HRMECs	Up	miR-205-5p/VEGF-A	Promotes angiogenesis	(98)
*HIF1A-AS2*	Preparation of peripheral blood mononuclear cells from NPDR patients and PDR patients	Up	VEGF	Promotes angiogenesis	(96)
*TDRG1*	HG-stimulated HRMECs	Up	miR-145/VEGF-A	Promotes ECs apoptosis, migration and enhanced permeability	(97)
*UCA1*	Plasma samples from DR patients HG-stimulated HRMECs	Up	miR‐624‐3p/VEGF-C	Promotes angiogenesis	(99)
*TUG1*	HG-stimulated HRMECs	Up	miR-145/VEGF-A	Promotes angiogenesis	(100)
*linc00174*	Vitreous humour from proliferative DR HG-stimulated HRMECs	Up	miR-150-5p/VEGF-A	Promotes angiogenesis	(101)

*HOTAIR*, HOX Transcript Antisense Intergenic RNA; *MALAT1*, Metastasis Associated Lung Adenocarcinoma Transcript 1; *HIF1A-AS2*, HIF1A antisense RNA 2; *TDRG1*, Testis Development Related Gene 1; *UCA1*, Urothelial Carcinoma-Associated 1; *TUG1*, Taurine-Upregulated Gene 1; HRMECs, human retinal microvascular endothelial cells; mRECs, mouse retinal microvascular endothelial cells; ECs, endothelial cells; DR, Diabetic retinopathy; VEGF, vascular endothelial growth factor; HIF-1α, hypoxia inducible factor 1 alpha; VH, vitreous humor; OIR, oxygen-induced retinopathy; NPDR, non-proliferative diabetic retinopathy; PDR, proliferative diabetic retinopathy.

## 5 Clinical application of lncRNAs in diabetic complications

LncRNAs are stable in a variety of body fluids, such as blood, plasma, serum, and urine, which can be used as a novel non-invasive biomarker for diabetic complications. Some lncRNAs have a diagnostic role in diabetic complications. *ANRIL* and *MALAT1* are upregulated in patients with DN, as the biomarker for the diagnosis of diabetic kidney disease (22, 26). *TINCR* and *HOTAIR* are downregulated in serum and myocardial biopsies of patients with DCM and can be used to effectively distinguish patients with DCM from healthy controls (58;102). LncRNAs are also known to act as prognostic molecules in DR. According to the receiver operating characteristic (ROC) curve, *MALAT1* and *HOTAIR* can be used as promising new biomarkers for predicting the severity of DR. Comparing NPDR with PDR patients, upregulation of serum *HOTAIR* and *MALAT1* was detected in PDR (103). Distinct lncRNA phenotype combinations may be able to discriminate DR patient sub-groups (NPDR and PDR). In the NPDR group, the most prevalent phenotype is *MIAT/WISPER/ZFAS1/H19*, while the prevalent lncRNA phenotypes in the PDR group is *HOTAIR/ANRIL/HULC/H19*. LncRNA variants may predict treatment outcomes. Following anti-VEGF therapy, DR patients with the *TUG1 A* or *MIAT T/C* exhibit worse therapeutic efficacy (104). Unfortunately, further validation in an expanded population is necessary due to the limited sample size included in this study.

Interestingly, lncRNAs are better suited as ideal candidates for therapeutic intervention because not encoding proteins. *TUG1* overexpression maintains mitochondrial morphology and dynamics in podocytes, silencing *KCNQ1OT1* alleviates myocardial dysfunction and attenuates myocardial fibrosis, targeting *AQP4-AS1* for the treatment of diabetic retinal neurovascular dysfunction, which is demonstrated in animal models (73, 89, 105). Nevertheless, for possible reasons such as off-target effects, adverse effects on cells other than those targeted, and lack of suitable delivery vehicles, the lack of lncRNA-based therapeutic approaches in human trials.

## 6 Conclusion

The regulatory role of lncRNAs in diabetic complications offers the possibility of finding new therapeutic targets. Interfering with the expression or function of lncRNAs, which involved in the diabetes-induced oxidative stress, apoptosis, and inflammation has the potential to improve the pathological process of diabetic complications. As mentioned above, *MALAT1* is strongly associated with the progression of DN, DR and DCM, as well as it is up-regulated in peripheral blood mononuclear cells (PBMCs) from type 2 diabetes patients. (106), suggesting that *MALAT1* is a crucial target molecule and biomarker for diabetic complications. Therefore, *MALAT1* should be investigated more deeply as an essential therapeutic target in future studies. Moreover, *TUG1* may be used as a therapeutic target for DN, *KCNQ1OT1* is specific for the interference of DCM, while *AQP4-AS1* for DR.

Current lncRNA targeting methods include the use of small interfering RNAs (siRNA), antisense oligonucleotides (ASOs), and the CRISPR/Cas9 system, which are delivered *in vivo* via a variety of vectors including viral vectors, liposomes, and exosomes. However, given the safety and delivery difficulties, CRISPR/Cas9 systems and viral vectors are more limited to basic research, and other approaches targeting lncRNAs also face a few concerns. The most significant issue is that the function and potential downstreams of the lncRNAs chosen to be targeted are still well understudied, and inadequate elucidation of their roles *in vivo*. Therefore, the use of lncRNA-targeted drugs in the clinic may face unintended consequences. In future, a better understanding of the mechanisms of lncRNA will pave the way for early diagnosis and the design of better treatments to reduce the morbidity and mortality of diabetic complications.

## Author contributions

MG: Investigation, Software, Writing – original draft. WL: Investigation, Software, Writing – original draft. JL: Resources, Writing – original draft. GY: Resources, Writing – original draft, Software. YT: Resources, Writing – original draft. XJ: Conceptualization, Writing – review & editing. YX: Conceptualization, Funding acquisition, Writing – review & editing.

## References

[1] Sun H Saeedi P Karuranga S Pinkepank M Ogurtsova K Duncan BB. IDF Diabetes Atlas: Global, regional and country-level diabetes prevalence estimates for 2021 and projections for 2045. Diabetes Res Clin Pract (2022) 183:109119. doi: 10.1016/j.diabres.2021.109119 34879977 PMC11057359

[2] Eizirik DL Pasquali L Cnop M. Pancreatic β-cells in type 1 and type 2 diabetes mellitus: different pathways to failure. Nat Rev Endocrinol (2020) 16(7):349–62. doi: 10.1038/s41574-020-0355-7 32398822

[3] Dagar N Das P Bisht P Taraphdar AK Velayutham R Arumugam S. Diabetic nephropathy: A twisted thread to unravel. Life Sci (2021) 278:119635. doi: 10.1016/j.lfs.2021.119635 34015285

[4] Lin YK Gao B Liu L Ang L Mizokami-Stout K Pop-Busui R. The Prevalence of Diabetic Microvascular Complications in China and the USA. Curr Diabetes Rep (2021) 21(6):16. doi: 10.1007/s11892-021-01387-3 33835284

[5] Gulsin GS Athithan L Mccann GP. Diabetic cardiomyopathy: prevalence, determinants and potential treatments. Ther Adv Endocrinol Metab (2019) 10:2042018819834869. doi: 10.1177/2042018819834869 30944723 PMC6437329

[6] Williams R Van Gaal L Lucioni C. Assessing the impact of complications on the costs of Type II diabetes. Diabetologia (2002). doi: 10.1007/s00125-002-0859-9 27942779

[7] Reddy MA Zhang E Natarajan R. Epigenetic mechanisms in diabetic complications and metabolic memory. Diabetologia (2015) 58(3):443–55. doi: 10.1007/s00125-014-3462-y PMC4324095 25481708

[8] Bridges MC Daulagala AC Kourtidis A. LNCcation: lncRNA localization and function. J Cell Biol (2021) 220(2):e202009045. doi: 10.1083/jcb.202009045 33464299 PMC7816648

[9] Gonzalez-Moro I Olazagoitia-Garmendia A Colli ML Cobo-Vuilleumier N Postler TS. Marselli L, et al. The T1D-associated lncRNA Lnc13 modulates human pancreatic β cell inflammation by allele-specific stabilization of STAT1 mRNA. Proc Natl Acad Sci U.S.A. (2020) 117(16):9022–31. doi: 10.1073/pnas.1914353117 PMC7183221 32284404

[10] González-Moro I Garcia-Etxebarria K Mendoza LM Fernández-Jiménez N Mentxaka J Olazagoitia-Garmendia A. LncRNA ARGI Contributes to Virus-Induced Pancreatic β Cell Inflammation Through Transcriptional Activation of IFN-Stimulated Genes. Adv Sci (Weinh) (2023) 10(25):e2300063. doi: 10.1002/advs.202300063 37382191 PMC10477904

[11] Guo WH Guo Q Liu YL Yan DD Jin L Zhang R. Mutated lncRNA increase the risk of type 2 diabetes by promoting β cell dysfunction and insulin resistance. Cell Death Dis (2022) 13(10):904. doi: 10.1038/s41419-022-05348-w 36302749 PMC9613878

[12] Chen J Ke S Zhong L Wu J Tseng A Morpurgo B. Long noncoding RNA MALAT1 regulates generation of reactive oxygen species and the insulin responses in male mice. Biochem Pharmacol (2018) 152:94–103. doi: 10.1016/j.bcp.2018.03.019 29577871 PMC12934558

[13] Persson F Rossing P. Diagnosis of diabetic kidney disease: state of the art and future perspective. Kidney Int Suppl (2011) 2018) 8(1):2–7. doi: 10.1016/j.kisu.2017.10.003 PMC6336222 30675433

[14] Cherney DZI Bakris GL. Novel therapies for diabetic kidney disease. Kidney Int Suppl (2011) 2018) 8(1):18–25. doi: 10.1016/j.kisu.2017.10.005 PMC6336219 30675435

[15] Liao L Chen J Zhang C Guo Y Liu W Liu W. LncRNA NEAT1 Promotes High Glucose-Induced Mesangial Cell Hypertrophy by Targeting miR-222-3p/CDKN1B Axis. Front Mol Biosci (2020) 7:627827. doi: 10.3389/fmolb.2020.627827 33585566 PMC7872960

[16] Huang S Xu Y Ge X Xu B Peng W Jiang X. Long noncoding RNA NEAT1 accelerates the proliferation and fibrosis in diabetic nephropathy through activating Akt/mTOR signaling pathway. J Cell Physiol (2019) 234(7):11200–7. doi: 10.1002/jcp.27770 30515796

[17] Wang X Xu Y Zhu YC Wang YK Li J Li XY. LncRNA NEAT1 promotes extracellular matrix accumulation and epithelial-to-mesenchymal transition by targeting miR-27b-3p and ZEB1 in diabetic nephropathy. J Cell Physiol (2019) 234(8):12926–33. doi: 10.1002/jcp.27959 PMC13483209 30549040

[18] Chang J Yu Y Fang Z He H Wang D Teng J. Long non-coding RNA CDKN2B-AS1 regulates high glucose-induced human mesangial cell injury *via* regulating the miR-15b-5p/WNT2B axis. Diabetol Metab Syndr (2020) 12(1):109. doi: 10.1186/s13098-020-00618-z 33298110 PMC7724838

[19] Li Y Zheng LL Huang DG Cao H Gao YH Fan ZC. LNCRNA CDKN2B-AS1 regulates mesangial cell proliferation and extracellular matrix accumulation *via* miR-424-5p/HMGA2 axis. BioMed Pharmacother (2020) 121:109622. doi: 10.1016/j.biopha.2019.109622 31707340

[20] Wang J Zhao SM. LncRNA-antisense non-coding RNA in the INK4 locus promotes pyroptosis *via* miR-497/thioredoxin-interacting protein axis in diabetic nephropathy. Life Sci (2021) 264:118728. doi: 10.1016/j.lfs.2020.118728 33160992

[21] Xiao M Bai S Chen J Li Y Zhang S Hu Z. CDKN2B-AS1 participates in high glucose-induced apoptosis and fibrosis *via* NOTCH2 through functioning as a miR-98-5p decoy in human podocytes and renal tubular cells. Diabetol Metab Syndr (2021) 13(1):107. doi: 10.1186/s13098-021-00725-5 34649592 PMC8518318

[22] Zhu Y Dai L Yu X Chen X Li Z Sun Y. Circulating expression and clinical significance of LncRNA ANRIL in diabetic kidney disease. Mol Biol Rep (2022) 49(11):10521–9. doi: 10.1007/s11033-022-07843-x PMC9618511 36129598

[23] Shen H Ming Y Xu C Xu Y Zhao S Zhang Q. Deregulation of long noncoding RNA (TUG1) contributes to excessive podocytes apoptosis by activating endoplasmic reticulum stress in the development of diabetic nephropathy. J Cell Physiol (2019). doi: 10.1002/jcp.28153 30671964

[24] Long J Galvan DL Mise K Kanwar YS Li L Poungavrin N. Role for carbohydrate response element-binding protein (ChREBP) in high glucose-mediated repression of long noncoding RNA Tug1. J Biol Chem (2020) 295(47):15840–52. doi: 10.1074/jbc.RA120.013228 PMC7681008 32467232

[25] Li L Long J Mise K Galvan DL Overbeek PA Tan L. PGC1α is required for the renoprotective effect of lncRNA Tug1 *in vivo* and links Tug1 with urea cycle metabolites. Cell Rep (2021) 36(6):109510. doi: 10.1016/j.celrep.2021.109510 34380028 PMC8369494

[26] Zhou LJ Yang DW Ou LN Guo XR Wu BL. Circulating Expression Level of LncRNA Malat1 in Diabetic Kidney Disease Patients and Its Clinical Significance. J Diabetes Res (2020) 2020:4729019. doi: 10.1155/2020/4729019 32832561 PMC7421584

[27] Petrica L Hogea E Gadalean F Vlad A Vlad M Dumitrascu V. Long noncoding RNAs may impact podocytes and proximal tubule function through modulating miRNAs expression in Early Diabetic Kidney Disease of Type 2 Diabetes Mellitus patients. Int J Med Sci (2021) 18(10):2093–101. doi: 10.7150/ijms.56551 PMC8040425 33859515

[28] Fawzy MS Abu Alsel BT Al Ageeli E Al-Qahtani SA Abdel-Daim MM Toraih EA. Long non-coding RNA MALAT1 and microRNA-499a expression profiles in diabetic ESRD patients undergoing dialysis: a preliminary cross-sectional analysis. Arch Physiol Biochem (2020) 126(2):172–82. doi: 10.1080/13813455.2018.1499119 30270667

[29] Hu M Wang R Li X Fan M Lin J Zhen J. LncRNA MALAT1 is dysregulated in diabetic nephropathy and involved in high glucose-induced podocyte injury *via* its interplay with β-catenin. J Cell Mol Med (2017) 21(11):2732–47. doi: 10.1111/jcmm.13189 PMC5661111 28444861

[30] Zuo Y Chen L He X Ye Z Li L Liu Z. Atorvastatin Regulates MALAT1/miR-200c/NRF2 Activity to Protect Against Podocyte Pyroptosis Induced by High Glucose. Diabetes Metab Syndr Obes (2021) 14:1631–45. doi: 10.2147/dmso.S298950 PMC8053520 33880049

[31] Zhang J Jiang T Liang X Shu S Xiang X Zhang W. lncRNA MALAT1 mediated high glucose-induced HK-2 cell epithelial-to-mesenchymal transition and injury. J Physiol Biochem (2019) 75(4):443–52. doi: 10.1007/s13105-019-00688-2 31388927

[32] Zhong W Zeng J Xue J Du A Xu Y. Knockdown of lncRNA PVT1 alleviates high glucose-induced proliferation and fibrosis in human mesangial cells by miR-23b-3p/WT1 axis. Diabetol Metab Syndr (2020) 12:33. doi: 10.1186/s13098-020-00539-x 32322310 PMC7161221

[33] Liu DW Zhang JH Liu FX Wang XT Pan SK Jiang DK. Silencing of long noncoding RNA PVT1 inhibits podocyte damage and apoptosis in diabetic nephropathy by upregulating FOXA1. . Exp Mol Med (2019) 51(8):1–15. doi: 10.1038/s12276-019-0259-6 PMC6802617 31371698

[34] Yang Y Lv X Fan Q Wang X Xu L Lu X. Analysis of circulating lncRNA expression profiles in patients with diabetes mellitus and diabetic nephropathy: Differential expression profile of circulating lncRNA. Clin Nephrol (2019) 92(1):25–35. doi: 10.5414/cn109525 31079598

[35] Li L Xu L Wen S Yang Y Li X Fan Q. The effect of lncRNA-ARAP1-AS2/ARAP1 on high glucose-induced cytoskeleton rearrangement and epithelial-mesenchymal transition in human renal tubular epithelial cells. J Cell Physiol (2020) 235(7-8):5787–95. doi: 10.1002/jcp.29512 31975379

[36] Zhu D Wu X Xue Q. Long non-coding RNA CASC2 restrains high glucose-induced proliferation, inflammation and fibrosis in human glomerular mesangial cells through mediating miR-135a-5p/TIMP3 axis and JNK signaling. Diabetol Metab Syndr (2021) 13(1):89. doi: 10.1186/s13098-021-00709-5 34446088 PMC8393478

[37] Yi H Peng R Zhang LY Sun Y Peng HM Liu HD. LincRNA-Gm4419 knockdown ameliorates NF-κB/NLRP3 inflammasome-mediated inflammation in diabetic nephropathy. Cell Death Dis (2017) 8(2):e2583. doi: 10.1038/cddis.2016.451 28151474 PMC5386454

[38] Mack M Yanagita M. Origin of myofibroblasts and cellular events triggering fibrosis. Kidney Int (2015) 87(2):297–307. doi: 10.1038/ki.2014.287 25162398

[39] Gao J Wang W Wang F Guo C. LncRNA-NR_033515 promotes proliferation, fibrogenesis and epithelial-to-mesenchymal transition by targeting miR-743b-5p in diabetic nephropathy. BioMed Pharmacother (2018) 106:543–52. doi: 10.1016/j.biopha.2018.06.104 29990842

[40] Nagai K Matsubara T Mima A Sumi E Kanamori H Iehara N. Gas6 induces Akt/mTOR-mediated mesangial hypertrophy in diabetic nephropathy. Kidney Int (2005) 68(2):552–61. doi: 10.1111/j.1523-1755.2005.00433.x 16014032

[41] Yang YL Xue M Jia YJ Hu F Zheng ZJ Wang L. Long noncoding RNA NEAT1 is involved in the protective effect of Klotho on renal tubular epithelial cells in diabetic kidney disease through the ERK1/2 signaling pathway. Exp Mol Med (2020) 52(2):266–80. doi: 10.1038/s12276-020-0381-5 PMC7062691 32054986

[42] Thomas AA Feng B Chakrabarti S. ANRIL regulates production of extracellular matrix proteins and vasoactive factors in diabetic complications. Am J Physiol Endocrinol Metab (2018) 314(3):E191–e200. doi: 10.1152/ajpendo.00268.2017 29118015

[43] Shen H Ming Y Xu C Xu Y Zhao S Zhang Q. Deregulation of long noncoding RNA (TUG1) contributes to excessive podocytes apoptosis by activating endoplasmic reticulum stress in the development of diabetic nephropathy. J Cell Physiol (2019) 234(9):15123–33. doi: 10.1002/jcp.28153 30671964

[44] Reiser J Kriz W Kretzler M Mundel P. The glomerular slit diaphragm is a modified adherens junction. J Am Soc Nephrol (2000) 11(1):1–8. doi: 10.1681/asn.V1111 10616834

[45] Millis MP Bowen D Kingsley C Watanabe RM Wolford JK. Variants in the plasmacytoma variant translocation gene (PVT1) are associated with end-stage renal disease attributed to type 1 diabetes. Diabetes (2007) 56(12):3027–32. doi: 10.2337/db07-0675 17881614

[46] Hanson RL Craig DW Millis MP Yeatts KA Kobes S Pearson JV. Identification of PVT1 as a candidate gene for end-stage renal disease in type 2 diabetes using a pooling-based genome-wide single nucleotide polymorphism association study. Diabetes (2007) 56(4):975–83. doi: 10.2337/db06-1072 17395743

[47] Yu D Yang X Zhu Y Xu F Zhang H Qiu Z. Knockdown of plasmacytoma variant translocation 1 (PVT1) inhibits high glucose-induced proliferation and renal fibrosis in HRMCs by regulating miR-23b-3p/early growth response factor 1 (EGR1). Endocr J (2021) 68(5):519–29. doi: 10.1507/endocrj.EJ20-0642 33408314

[48] Rubler S Dlugash J Yuceoglu YZ Kumral T Branwood AW Grishman A. New type of cardiomyopathy associated with diabetic glomerulosclerosis. Am J Cardiol (1972) 30(6):595–602. doi: 10.1016/0002-9149(72)90595-4 4263660

[49] Jia G Hill MA Sowers JR. Diabetic Cardiomyopathy: An Update of Mechanisms Contributing to This Clinical Entity. Circ Res (2018) 122(4):624–38. doi: 10.1161/circresaha.117.311586 PMC5819359 29449364

[50] Wei J Zhao Y Liang H Du W Wang L. Preliminary evidence for the presence of multiple forms of cell death in diabetes cardiomyopathy. Acta Pharm Sin B (2022) 12(1):1–17. doi: 10.1016/j.apsb.2021.08.026 35127369 PMC8799881

[51] Wang C Liu G Yang H Guo S Wang H Dong Z. MALAT1-mediated recruitment of the histone methyltransferase EZH2 to the microRNA-22 promoter leads to cardiomyocyte apoptosis in diabetic cardiomyopathy. Sci Total Environ (2021) 766:142191. doi: 10.1016/j.scitotenv.2020.142191 33097254

[52] Zhou X Zhang W Jin M Chen J Xu W Kong X. lncRNA MIAT functions as a competing endogenous RNA to upregulate DAPK2 by sponging miR-22-3p in diabetic cardiomyopathy. Cell Death Dis (2017) 8(7):e2929. doi: 10.1038/cddis.2017.321 28703801 PMC5550866

[53] Zhang J Zhang M Yang Z Huang S Wu X Cao L. PDCD4 deficiency ameliorates left ventricular remodeling and insulin resistance in a rat model of type 2 diabetic cardiomyopathy. BMJ Open Diabetes Res Care (2020) 8(1):e001081. doi: 10.1136/bmjdrc-2019-001081 PMC7228667 32371529

[54] Lu K Chen Q Li M He L Riaz F Zhang T. Programmed cell death factor 4 (PDCD4), a novel therapy target for metabolic diseases besides cancer. Free Radic Biol Med (2020) 159:150–63. doi: 10.1016/j.freeradbiomed.2020.06.016 32745771

[55] Zhao SF Ye YX Xu JD He Y Zhang DW Xia ZY. Long non-coding RNA KCNQ1OT1 increases the expression of PDCD4 by targeting miR-181a-5p, contributing to cardiomyocyte apoptosis in diabetic cardiomyopathy. Acta Diabetol (2021) 58(9):1251–67. doi: 10.1007/s00592-021-01713-x 33907874

[56] Zhang Q Li D Dong X Zhang X Liu J Peng L. LncDACH1 promotes mitochondrial oxidative stress of cardiomyocytes by interacting with sirtuin3 and aggravates diabetic cardiomyopathy. . Sci China Life Sci (2022) 65(6):1198–212. doi: 10.1007/s11427-021-1982-8 34668131

[57] Gao L Wang X Guo S Xiao L Liang C Wang Z. LncRNA HOTAIR functions as a competing endogenous RNA to upregulate SIRT1 by sponging miR-34a in diabetic cardiomyopathy. J Cell Physiol (2019) 234(4):4944–58. doi: 10.1002/jcp.27296 30216438

[58] Qi K Zhong J. LncRNA HOTAIR improves diabetic cardiomyopathy by increasing viability of cardiomyocytes through activation of the PI3K/Akt pathway. Exp Ther Med (2018) 16(6):4817–23. doi: 10.3892/etm.2018.6755 PMC6257662 30542437

[59] Wang S Duan J Liao J Wang Y Xiao X Li L. LncRNA H19 inhibits ER stress induced apoptosis and improves diabetic cardiomyopathy by regulating PI3K/AKT/mTOR axis. Aging (2022) 14(16):6809–28. doi: 10.18632/aging.204256 PMC9467416 36044268

[60] Cai X Cullen BR. The imprinted H19 noncoding RNA is a primary microRNA precursor. RNA (2007) 13(3):313–6. doi: 10.1261/rna.351707 PMC1800509 17237358

[61] Li X Wang H Yao B Xu W Chen J Zhou X. lncRNA H19/miR-675 axis regulates cardiomyocyte apoptosis by targeting VDAC1 in diabetic cardiomyopathy. Sci Rep (2016) 6:36340. doi: 10.1038/srep36340 27796346 PMC5087087

[62] Ghosh R Pattison JS. Macroautophagy and Chaperone-Mediated Autophagy in Heart Failure: The Known and the Unknown. Oxid Med Cell Longev (2018) 2018:8602041. doi: 10.1155/2018/8602041 29576856 PMC5822756

[63] Wu QQ Liu C Cai Z Xie Q Hu T Duan M. High-mobility group AT-hook 1 promotes cardiac dysfunction in diabetic cardiomyopathy *via* autophagy inhibition. Cell Death Dis (2020) 11(3):160. doi: 10.1038/s41419-020-2316-4 32123163 PMC7052237

[64] Chen D Zhang M. GAS5 regulates diabetic cardiomyopathy *via* miR−221−3p/p27 axis−associated autophagy. Mol Med Rep (2021) 23(2):135. doi: 10.3892/mmr.2020.11774 33313941 PMC7751493

[65] Feng Y Xu W Zhang W Wang W Liu T Zhou X. LncRNA DCRF regulates cardiomyocyte autophagy by targeting miR-551b-5p in diabetic cardiomyopathy. Theranostics (2019) 9(15):4558–66. doi: 10.7150/thno.31052 PMC6599651 31285779

[66] Hu X Sui X Li L Huang X Rong R Su X. Protocadherin 17 acts as a tumour suppressor inducing tumour cell apoptosis and autophagy, and is frequently methylated in gastric and colorectal cancers. J Pathol (2013) 229(1):62–73. doi: 10.1002/path.4093 22926751

[67] Wu JC Wang FZ Tsai ML Lo CY Badmaev V Ho CT. Se-Allylselenocysteine induces autophagy by modulating the AMPK/mTOR signaling pathway and epigenetic regulation of PCDH17 in human colorectal adenocarcinoma cells. Mol Nutr Food Res (2015) 59(12):2511–22. doi: 10.1002/mnfr.201500373 26395119

[68] Zhuo C Jiang R Lin X Shao M. LncRNA H19 inhibits autophagy by epigenetically silencing of DIRAS3 in diabetic cardiomyopathy. Oncotarget (2017) 8(1):1429–37. doi: 10.18632/oncotarget.13637 PMC5352066 27903964

[69] Ketelut-Carneiro N Fitzgerald KA. Apoptosis, Pyroptosis, and Necroptosis-Oh My! The Many Ways a Cell Can Die. J Mol Biol (2022) 434(4):167378. doi: 10.1016/j.jmb.2021.167378 34838807

[70] Fang Y Tian S Pan Y Li W Wang Q Tang Y. Pyroptosis: A new frontier in cancer. BioMed Pharmacother (2020) 121:109595. doi: 10.1016/j.biopha.2019.109595 31710896

[71] Meng L Lin H Huang X Weng J Peng F Wu S. METTL14 suppresses pyroptosis and diabetic cardiomyopathy by downregulating TINCR lncRNA. Cell Death Dis (2022) 13(1):38. doi: 10.1038/s41419-021-04484-z 35013106 PMC8748685

[72] Xu Y Fang H Xu Q Xu C Yang L Huang C. LncRNA GAS5 inhibits NLRP3 inflammasome activation-mediated pyroptosis in diabetic cardiomyopathy by targeting miR-34b-3p/AHR. Cell Cycle (2020) 19(22):3054–65. doi: 10.1080/15384101.2020.1831245 PMC7714483 33092444

[73] Yang F Qin Y Lv J Wang Y Che H Chen X. Silencing long non-coding RNA Kcnq1ot1 alleviates pyroptosis and fibrosis in diabetic cardiomyopathy. Cell Death Dis (2018) 9(10):1000. doi: 10.1038/s41419-018-1029-4 30250027 PMC6155223

[74] Xiao W Zheng D Chen X Yu B Deng K Ma J. Long non-coding RNA MIAT is involved in the regulation of pyroptosis in diabetic cardiomyopathy *via* targeting miR-214-3p. iScience (2021) 24(12):103518. doi: 10.1016/j.isci.2021.103518 34950859 PMC8671938

[75] Liu M López De Juan Abad B Cheng K. Cardiac fibrosis: Myofibroblast-mediated pathological regulation and drug delivery strategies. Adv Drug Delivery Rev (2021) 173:504–19. doi: 10.1016/j.addr.2021.03.021 PMC8299409 33831476

[76] Che H Wang Y Li H Li Y Sahil A Lv J. Melatonin alleviates cardiac fibrosis *via* inhibiting lncRNA MALAT1/miR-141-mediated NLRP3 inflammasome and TGF-β1/Smads signaling in diabetic cardiomyopathy. FASEB J (2020) 34(4):5282–98. doi: 10.1096/fj.201902692R 32067273

[77] Zhang Y Zhang YY Li TT Wang J Jiang Y Zhao Y. Ablation of interleukin-17 alleviated cardiac interstitial fibrosis and improved cardiac function *via* inhibiting long non-coding RNA-AK081284 in diabetic mice. J Mol Cell Cardiol (2018) 115:64–72. doi: 10.1016/j.yjmcc.2018.01.001 29305939

[78] Qi Y Wu H Mai C Lin H Shen J Zhang X. LncRNA-MIAT-Mediated miR-214-3p Silencing Is Responsible for IL-17 Production and Cardiac Fibrosis in Diabetic Cardiomyopathy. Front Cell Dev Biol (2020) 8:243. doi: 10.3389/fcell.2020.00243 32351959 PMC7174588

[79] Zheng D Zhang Y Hu Y Guan J Xu L Xiao W. Long noncoding RNA Crnde attenuates cardiac fibrosis *via* Smad3-Crnde negative feedback in diabetic cardiomyopathy. FEBS J (2019) 286(9):1645–55. doi: 10.1111/febs.14780 PMC6849551 30748104

[80] Tan TE Wong TY. Diabetic retinopathy: Looking forward to 2030. Front Endocrinol (Lausanne) (2022) 13:1077669. doi: 10.3389/fendo.2022.1077669 36699020 PMC9868457

[81] Yau JW Rogers SL Kawasaki R Lamoureux EL Kowalski JW Bek T. Global prevalence and major risk factors of diabetic retinopathy. Diabetes Care (2012) 35(3):556–64. doi: 10.2337/dc11-1909 PMC3322721 22301125

[82] Couturier A Rey PA Erginay A Lavia C Bonnin S Dupas B. Widefield OCT-Angiography and Fluorescein Angiography Assessments of Nonperfusion in Diabetic Retinopathy and Edema Treated with Anti-Vascular Endothelial Growth Factor. Ophthalmology (2019) 126(12):1685–94. doi: 10.1016/j.ophtha.2019.06.022 31383483

[83] Carpi-Santos R De Melo Reis RA Gomes FCA Calaza KC. Contribution of Müller Cells in the Diabetic Retinopathy Development: Focus on Oxidative Stress and Inflammation. Antioxidants (Basel) (2022) 11(4):617. doi: 10.3390/antiox11040617 35453302 PMC9027671

[84] Yang S Qi S Wang C. The role of retinal Müller cells in diabetic retinopathy and related therapeutic advances. Front Cell Dev Biol (2022) 10:1047487. doi: 10.3389/fcell.2022.1047487 36531955 PMC9757137

[85] Zhang J Chen C Wu L Wang Q Chen J Zhang S. C-myc contributes to the release of Müller cells-derived proinflammatory cytokines by regulating lncRNA MIAT/XNIP pathway. Int J Biochem Cell Biol (2019) 114:105574. doi: 10.1016/j.biocel.2019.105574 31344482

[86] Fu S Zheng Y Sun Y Lai M Qiu J Gui F. Suppressing long noncoding RNA OGRU ameliorates diabetic retinopathy by inhibition of oxidative stress and inflammation *via* miR-320/USP14 axis. Free Radic Biol Med (2021) 169:361–81. doi: 10.1016/j.freeradbiomed.2021.03.016 33762162

[87] Yao J Wang XQ Li YJ Shan K Yang H Wang YN. Long non-coding RNA MALAT1 regulates retinal neurodegeneration through CREB signaling. EMBO Mol Med (2016) 8(4):346–62. doi: 10.15252/emmm.201505725 PMC4818754 26964565

[88] Verkman AS Anderson MO Papadopoulos MC. Aquaporins: important but elusive drug targets. Nat Rev Drug Discov (2014) 13(4):259–77. doi: 10.1038/nrd4226 PMC4067137 24625825

[89] Li X Zhu J Zhong Y Liu C Yao M Sun Y. Targeting long noncoding RNA-AQP4-AS1 for the treatment of retinal neurovascular dysfunction in diabetes mellitus. EBioMedicine (2022) 77:103857. doi: 10.1016/j.ebiom.2022.103857 35172268 PMC8850682

[90] Navaratna D Mcguire PG Menicucci G Das A. Proteolytic degradation of VE-cadherin alters the blood-retinal barrier in diabetes. Diabetes (2007) 56(9):2380–7. doi: 10.2337/db06-1694 17536065

[91] Biswas S Feng B Chen S Liu J Aref-Eshghi E Gonder J. The Long Non-Coding RNA HOTAIR Is a Critical Epigenetic Mediator of Angiogenesis in Diabetic Retinopathy. Invest Ophthalmol Vis Sci (2021) 62(3):20. doi: 10.1167/iovs.62.3.20 PMC7980040 33724292

[92] Zhao D Zhao Y Wang J Wu L Liu Y Zhao S. Long noncoding RNA Hotair facilitates retinal endothelial cell dysfunction in diabetic retinopathy. Clin Sci (Lond) (2020) 134(17):2419–34. doi: 10.1042/cs20200694 32812634

[93] Liu P Jia SB Shi JM Li WJ Tang LS Zhu XH. LncRNA-MALAT1 promotes neovascularization in diabetic retinopathy through regulating miR-125b/VE-cadherin axis. Biosci Rep (2019) 39(5):BSR20181469. doi: 10.1042/bsr20181469 30988072 PMC6522718

[94] Haidari M Zhang W Willerson JT Dixon RA. Disruption of endothelial adherens junctions by high glucose is mediated by protein kinase C-β-dependent vascular endothelial cadherin tyrosine phosphorylation. Cardiovasc Diabetol (2014) 13:105. doi: 10.1186/1475-2840-13-105 25927959 PMC4223716

[95] Sehgal P Mathew S Sivadas A Ray A Tanwar J Vishwakarma S. LncRNA VEAL2 regulates PRKCB2 to modulate endothelial permeability in diabetic retinopathy. EMBO J (2021) 40(15):e107134. doi: 10.15252/embj.2020107134 34180064 PMC8327952

[96] Atef MM Shafik NM Hafez YM Watany MM Selim A Shafik HM. The evolving role of long noncoding RNA HIF1A-AS2 in diabetic retinopathy: a cross-link axis between hypoxia, oxidative stress and angiogenesis *via* MAPK/VEGF-dependent pathway. Redox Rep (2022) 27(1):70–8. doi: 10.1080/13510002.2022.2050086 PMC8928809 35285425

[97] Gong Q Dong W Fan Y Chen F Bian X Xu X. LncRNA TDRG1-Mediated Overexpression of VEGF Aggravated Retinal Microvascular Endothelial Cell Dysfunction in Diabetic Retinopathy. Front Pharmacol (2019) 10:1703. doi: 10.3389/fphar.2019.01703 32082175 PMC7005225

[98] Tan A Li T Ruan L Yang J Luo Y Li L. Knockdown of Malat1 alleviates high-glucose-induced angiogenesis through regulating miR-205-5p/VEGF-A axis. Exp Eye Res (2021) 207:108585. doi: 10.1016/j.exer.2021.108585 33887222

[99] Yan H Yao P Hu K Li X Li H. Long non-coding ribonucleic acid urothelial carcinoma-associated 1 promotes high glucose-induced human retinal endothelial cells angiogenesis through regulating micro-ribonucleic acid-624-3p/vascular endothelial growth factor C. J Diabetes Investig (2021) 12(11):1948–57. doi: 10.1111/jdi.13617 PMC8565426 34137197

[100] Shi Q Tang J Wang M Xu L Shi L. Knockdown of Long Non-coding RNA TUG1 Suppresses Migration and Tube Formation in High Glucose-Stimulated Human Retinal Microvascular Endothelial Cells by Sponging miRNA-145. Mol Biotechnol (2022) 64(2):171–7. doi: 10.1007/s12033-021-00398-5 34554391

[101] Wang JJ Wu KF Wang DD. A novel regulatory network of linc00174/miR-150-5p/VEGFA modulates pathological angiogenesis in diabetic retinopathy. Can J Physiol Pharmacol (2021) 99(11):1175–83. doi: 10.1139/cjpp-2021-0036 34081870

[102] Chen Y Tan S Liu M Li J. LncRNA TINCR is downregulated in diabetic cardiomyopathy and relates to cardiomyocyte apoptosis. Scand Cardiovasc J (2018) 52(6):335–9. doi: 10.1080/14017431.2018.1546896 30453794

[103] Shaker OG Abdelaleem OO Mahmoud RH Abdelghaffar NK Ahmed TI Said OM. Diagnostic and prognostic role of serum miR-20b, miR-17-3p, HOTAIR, and MALAT1 in diabetic retinopathy. IUBMB Life (2019) 71(3):310–20. doi: 10.1002/iub.1970 30468285

[104] Mohammad HMF Abdelghany AA Al Ageeli E Kattan SW Hassan R Toraih EA. Long Non-Coding RNAs Gene Variants as Molecular Markers for Diabetic Retinopathy Risk and Response to Anti-VEGF Therapy. Pharmgenomics Pers Med (2021) 14:997–1014. doi: 10.2147/pgpm.S322463 34429633 PMC8374537

[105] Long J Badal SS Ye Z Wang Y Ayanga BA Galvan DL. Long noncoding RNA Tug1 regulates mitochondrial bioenergetics in diabetic nephropathy. J Clin Invest (2016) 126(11):4205–18. doi: 10.1172/jci87927 PMC5096930 27760051

[106] Sathishkumar C Prabu P Mohan V Balasubramanyam M. Linking a role of lncRNAs (long non-coding RNAs) with insulin resistance, accelerated senescence, and inflammation in patients with type 2 diabetes. Hum Genomics (2018) 12(1):41. doi: 10.1186/s40246-018-0173-3 30139387 PMC6107963

